# Links between Belowground and Aboveground Resource-Related Traits Reveal Species Growth Strategies that Promote Invasive Advantages

**DOI:** 10.1371/journal.pone.0104189

**Published:** 2014-08-08

**Authors:** Maria S. Smith, Jason D. Fridley, Marc Goebel, Taryn L. Bauerle

**Affiliations:** 1 Department of Horticulture, Cornell University, 134A Plant Science Building, Ithaca, New York, United States of America; 2 Department of Biology, Syracuse University, Syracuse, New York, United States of America; 3 Department of Natural Resources, Cornell University, Ithaca, New York, United States of America; Fudan University, China

## Abstract

Belowground processes are rarely considered in comparison studies of native verses invasive species. We examined relationships between belowground fine root production and lifespan, leaf phenology, and seasonal nitrogen dynamics of *Lonicera japonica* (non-native) versus *L. sempervirens* (native) and *Frangula alnus* (non-native) versus *Rhamnus alnifolia* (native), over time. First and second order fine roots were monitored from 2010 to 2012 using minirhizotron technology and rhizotron windows. ^15^N uptake of fine roots was measured across spring and fall seasons. Significant differences in fine root production across seasons were seen between *Lonicera* species, but not between *Frangula* and *Rhamnus*, with both groups having notable asynchrony in regards to the timing of leaf production. Root order and the number of root neighbors at the time of root death were the strongest predictors of root lifespan of both species pairs. Seasonal ^15^N uptake was higher in spring than in the fall, which did not support the need for higher root activity to correspond with extended leaf phenology. We found higher spring ^15^N uptake in non-native *L. japonica* compared to native *L. sempervirens*, although there was no difference in ^15^N uptake between *Frangula* and *Rhamnus* species. Our findings indicate the potential for fast-growing non-native *Lonicera japonica* and *Frangula alnus* to outcompete native counterparts through differences in biomass allocation, root turnover, and nitrogen uptake, however evidence that this is a general strategy of invader dominance is limited.

## Introduction

Non-native plant species have the potential to significantly alter both community composition and ecosystem processes such as net primary productivity, nutrient cycling, and water availability [1]–[3], and are one of the largest contributors to global biodiversity loss [4]. Recently, many efforts have focused on identifying plant traits to understand the underlying process of plant invasions, including differentials in growth rate and size, reproduction rate, and physiological traits inherent to successful invaders [5], [6]. However, these studies are limited in the use of aboveground plant traits.

An important factor contributing to the success of non-native plant species is how they capture resources, including the timing of leaf emergence and senescence. In deciduous forests, growth phenology exerts strong control over aboveground plant resource acquisition and nutrient cycling through increased aboveground foliage production [7], [8]. It was recently demonstrated that a broad range of non-native species exhibit extended leaf phenology, capturing carbon earlier in the spring and particularly later in the fall, an uncommon attribute in native species [9]. Along with contrasting phenology, many fast-growing, non-native species exhibit leaf-level traits such as high specific leaf area (SLA) and higher foliar nitrogen and phosphorous content [5], [10]. These traits typically correspond to faster plant growth strategies and are negatively correlated with leaf lifespan (LL) [11], [12].

The capacity for high plant relative growth rate (RGR) is often credited for the success of non-native woody plants during the establishment phase following colonization [13]–[15], particularly under high-resource, low-stress conditions [16]. Invasion in low-resource environments also occurs, though with less frequency than high-resource environments [17], 18]. To effectively compete in areas where native plants have evolved to efficiently utilize limited resources, fast-growing non-native plants must either persist in the environment through resource use efficiency adjustment [10], [18], increasing resource availability through N-fixation [19] or positive-feedback through litter decomposition [20]–[23]. Additionally, many aboveground traits generally common to fast-growing non-natives exhibit morphological and/or physiological plastic responses to fluxes in light availability [24], [25]. Despite the importance in resource acquisition and nutrient cycling are analogous morphological and physiological traits of the finest ephemeral roots in native and non-native plants, which are much less understood [26], [27].

The finest, most distal root order branches in a root system are responsible for water and nutrient acquisition and are at variable rates of decomposition a source of carbon (C) and nutrients for the soil system [28]–[30]. In contrast with higher order structural roots, low-order fine roots are characterized by higher N concentration, higher specific root length (SRL), and higher respiration rates [31]–[33]. Production and lifespan of these low-order roots are controlled by both endogenous factors (diameter, mycorrhizal associations) [34], [35] and environmental factors (temperature, soil moisture, nutrient availability and rooting depth), though the importance of these factors varies considerably over species and environment [35]–[38].

Although controls on leaf phenology are well understood [39], we have much less knowledge of controls on root phenology and the relationship between root and leaf phenology. Previous studies on temperate tree species found root production and mortality are highly synchronized with foliar production, where root systems are expanded prior to leaf growth in order to support necessary water and nutrient uptake [36], [40], [41]. However, periodicity of root production is strongly contingent upon environmental conditions. In Northern hardwood forests under unlimited water conditions, fine root production has been shown to occur slowly in spring and peak mid-summer before declining in fall [37], [42], [43], whereas peak fine root production generally occurs in spring and/or fall under late-summer water limitation [44], [45]. Both root production and mortality are usually low over winter due to frozen soils that prohibit water uptake and microbial activity [37]. Understanding environmental controls on root phenology is necessary to predict how introduced non-native species may respond to new environmental conditions.

Several attempts have been made to link leaf and root phenology and lifespan. A relationship was found between the production of elongating roots (root elongation intensity – REI) with leaf phenology, where the highest REI levels coincided with active foliage expansion and growth in mature oak trees between April and early August [45]. Likewise, a negative correlation was found in the timing of peak leaf and root production in aspen forests, with peak root production occurring 45 days after leaf production [46]. Withington *et al*. [47] compared lifespans of roots and leaves among species of varying growth rates, and found no linkage in longevity between leaves and roots. However, a significant correlation between root N concentration, root N:C, and root longevity was found, supporting evidence for a linkage between root traits and lifespan. This linkage was further supported with correlations of various root traits to median lifespan across 12 species of temperate trees, with diameter, calcium content and tree wood density positively correlating with lifespan, SRL, and N:C ratio, and plant growth negatively correlating with lifespan [38]. For non-native species, precise timing of fine root production during early spring has important implications for potential co-opting of ephemeral resources by early leafing invaders, while extended root production into autumn months could explain continued support for extended leaf production. Additionally, knowledge of root lifespan can be coupled to aboveground traits of non-native plants for greater precision in predicting non-native potential for invasive growth and their ability to alter ecosystem processes.

In this study, we explored fine root and leaf production, their life spans, and fine root seasonal nitrogen uptake of the Eastern U.S. native forest understory species *Lonicera sempervirens* L. (vine; Caprifoliaceae) and *Rhamnus alnifolia* L'Hér. (shrub; Rhamnaceae), and their non-native invasive congeners *Lonicera japonica* Thunb. and *Frangula alnus* Mill. These four species demonstrated strong contrasts in leaf phenology and lifespan among over 70 species from seven different genera in a common garden study [9]. We examined the relationship of aboveground to belowground growth phenology between native and non-native pairs, with the expectation that non-natives exhibit patterns of root production corresponding with aboveground leaf phenology. In addition, we expected non-native species would have shorter-lived roots compared to native species, the latter showing longer root lifespan for roots born during the spring season. We expected root order to be a more significant predictor of root life span, rather than root diameter, such that higher order root branches have longer lifespans than lower order branches, as suggested by previous studies [35], [38]. Lastly, we compared root nitrogen uptake, using isotopic ^15^N, over fall and spring seasons between congeneric pairs, expecting a difference in root physiological activity between natives and non-natives to support extended leaf phenology, with non-native plants having faster N uptake over natives.

## Materials and Methods

### Leaf phenology

Three replicate individuals of *L. japonica*, *L. sempervirens*, *F. alnus*, and *R. alnifolia* were established between 2006 and 2007 in a common garden plot in Syracuse, New York (USA) 43°03' N, 76°09' W, no specific permissions were required for these locations/activities nor did they involve endangered or protected species. Individuals were maintained under 80% shade from mid-May through mid-October, simulating canopy closure and understory light conditions. Leaf phenology and demography was collected biweekly over the 2008 through 2010 growing seasons, by following five branches per individual monitored for the timing of spring foliar bud and leaf development and biweekly leaf production counts [9]. From the three years of data collection, 2008 data was used for displaying phenology and lifespan. Data collected from 2008 and 2009 were very similar, while phenology data for 2010 showed much earlier full leaf emergence due to a warmer than average spring [9]. Demography data, however, did not differ between the three measured years. Leaf lifespan was measured as an average across the growing season, calculated as the area beneath the total live leaves curve divided by the total number of emerged leaves [48] (Fig. S1).

### Root production and lifespan

*L. japonica, L. sempervirens* and *F. alnus* were propagated using 0.8% IBA in talc (Hormodin® 3, OHP Inc.) from hardwood cuttings of established individuals in November 2009. Cuttings of *R. alnifolia* were obtained from Reeseville Ridge Nursery, Reeseville, WI due to lack of sufficient propagation material. In May 2010, seven replicates of each species were transferred to 18-gallon pots filled with a 50–50 mix of perlite and sterilized Hudson silty clay loam soil (pH 7.5) excavated from the Blue Grass Lane field site in Ithaca, NY, 42°27'33" N, 76°27'45" W, no specific permissions were required for these locations/activities nor did they involve endangered or protected species. All pots contained replaceable mylar observation ‘windows’ (250×336 mm) to provide access to undisturbed attached roots. Aluminum insulation was used to cover the windows to prevent light penetration into the root box.

Plants were arranged in a completely randomized design. During periods of full forest canopy enclosure, from mid-May through mid-October, plants were maintained under knitted black 80% shade cloth (Dewitt Company Inc., Sikeston, MO, USA) to mimic natural growing conditions. Plants were overwintered from December to March to prevent frost damage, and watered as needed during summer months.

### Root observations and measurements

Acrylic minirhizotron root observation tubes (50.5 cm long, 6 cm outer diameter) were installed in May 2010 at a 30° angle from the vertical. Two minirhizotron tubes were installed per box, equidistant from each side of the plant. Each species had a total of 14 observation tubes, for 56 tubes total. Ten centimeters of each tube was left above the soil surface, wrapped in black electrical tape and capped with a rubber stopper to prevent light penetration. White metal cans were placed over the top of the tube to minimize radiant heating, and PVC plastic plugs were used to prevent water from entering the bottom of the tubes.

Root image observations were collected along each tube in one-week intervals during the growing season (May through September) and once monthly during the dormant periods (October through April) using Bartz I-CAP image capture system (Bartz Technology, Santa Barbara, CA, USA). Captured images measured 14 mm in height and 18 mm in width. All images were analyzed for root population counts, seasonal root production, and survivorship using WinRhizo Tron MF (Regents Inc., Quebec, Canada). Root births and root deaths were estimated by calculating the date midway between the birth observation date and death and the previous observation date. Root death was as a root that had disappeared from the viewing area and did not reappear. A root was considered censored (i.e. incomplete information) if it was not dead at the end of the observation period or had disappeared from the viewing area. Roots appearing in continuous frames within the same tube were only counted once. A topological approach was applied to classifying root order, where roots terminating in a meristem, identified as a root tip, with no dependent laterals were considered first order, roots with only one lateral were considered second order, and so forth [32], [49]. Only first and second order roots were counted due to the low sample size of third order roots (less than 10) visible for each species.

Differences in root lifespan were analyzed using Cox proportional hazards regression (PROC PHREG, Enterprise Guide 5.1, SAS Institute, Inc., Cary, NC, US). Cox proportional hazards allow the evaluation of each covariate while controlling for the effects of other covariates [50]. In this analysis, individual roots are evaluated for their ‘hazard’, the risk of mortality of a root over time *t*, where *t* is the product of a baseline hazard function of *k* covariates [51]. The partial likelihood method from PROC PHREG estimates a β coefficient for each covariate [50], and the model calculates a chi-square statistic to test the null hypothesis that each β is equal to zero. Parameter estimates can be positive or negative, *e.g*. a negative β indicating a decreased hazard of mortality (longer root lifespan) as the covariate increases [35], [52]. Covariates in the root survivorship model included root diameter and root neighbors (the number of roots present in the image window), and root order. Roots were additionally analyzed for differences in survivorship by season of birth. Roots born during winter months were excluded from the analysis and the remaining roots were pooled due to insufficient number of roots. The hazard ratio (e^β^) was interpreted based on classification of covariates as either categorical or continuous [35], [51]. For example, the hazard ratio of a categorical covariate such as “root order” would be interpreted as the ratio of the hazard of a first order root (1) to that of a second order root (2), controlling for all other covariates. For a quantitative covariate such as root diameter, the estimated percent change in the hazard associated with one unit of change in the covariate would be one subtracted from the hazard ratio and multiplied by 100 [51]. Seasonal root production, categorized by meteorological seasons, was calculated as the percent of fine roots produced by each species seasonally as a proportion of the total. Standing crop biomass was calculated as the difference between cumulative production and cumulative mortality of fine roots.

### ^15^N Uptake experiment

Seasonal individual fine root nitrogen uptake was measured using the ^15^N tracer method in October 2010 and May 2011. Root growth was tracked daily for four weeks prior to ^15^N sampling using different colored paint markers to ensure precise root age. New white root tips less than three days old without lateral branching were used to track declining nitrate uptake with increasing root age (as in [53]).

Prior to measurements, small cuts were applied to mylar windows to access root tips and keep them intact as well as to clean the tips from soil particles. The lid of the Eppendorf tubes was punctured for root insertion and to minimize solution evaporation from the tube. Root tips were placed overnight into a buffer solution of 0.6 mL with a pH 5.7, containing 0.5 mL of 1 mM, unlabeled KNO3 buffered with 10 mM MES, 1 mM CaSO_4_, and 4 uM K_2_HPO_4_ [53]. Three to four new fine roots were used from each plant to test for nitrate uptake at three different time periods (zero, three, six hours), using labeled K^15^NO_3_ (98 At %, Sigma Aldrich, St. Louis, MO, USA), as well as an unlabeled KNO_3_ as control. The following day, roots were placed into a new vial containing 1 mM labeled K^15^NO_3_ for zero, three or six hour time periods. All controls were also transferred to fresh tubes containing the 1 mM unlabeled buffer solution for six hours. The mylar windows on the front of the root box remained covered, with the majority of root still in the soil, with the exception of the portion of roots exposed to the tube. At the end of the zero, three, and six hour time period, roots were excised, rinsed in deionized water and placed into aluminum tins of a 96-well plate to dry at 60°C for 12 hours. Dried tissue samples were weighed and analyzed for δ^15^N enrichment using an elemental analyzer (EA-IRMS) at the Cornell University Stable Isotope Laboratory (COIL, Ithaca, NY). Gross N-uptake was calculated from the % ^15^N in root tissue, expressed as in µg of ^15^N per µg of root tissue. Using pooled root data for each species, a mixed-effects model was constructed to test the effects of hours of ^15^N exposure, season, native status and interactions on fine root ^15^N concentrations in JMP (SAS Institute Inc, Cary, NC, v. 10.0). Additionally, individual models were constructed to test for effects of time of native status within individual seasons.

## Results

### Leaf phenology and demography

Full leaf expansion of non-native *L. japonica* occurred eight days prior to *L. sempervirens*, while 90% of leaf fall occurred for *L. japonica* five days prior to *L. sempervirens*, (Fig. S1). The two *Lonicera* species had large differences in leaf life span, with an average of 38 days for *L. japonica* compared to 120 days for *L. sempervirens* (data not shown). The mean total leaves produced per new annual shoot by *L. japonica* (257 leaves) significantly exceeded those of *L. sempervirens* (31 leaves, *P* = 0.0048),

*Frangula* and *Rhamnus* species had a larger contrast in fall leaf retention compared to *Lonicera* species. While the date of full leaf expansion within *Frangula* and *Rhamnus* was the same for both species, April 23rd, the date of 90% leaf drop was approximately one month later for non-native *F. alnus*, occurring on November 11th compared to October 9th for native *R. alnifolia*. Leaf life span was similar between *Frangula* and *Rhamnus* at 114 days in *F. alnus* and 110 days in *R. alnifolia* (data not shown). There was no difference in total leaf production per shoot between non-native *F. alnus* and native *R. alnifolia* (14 leaves and 9 leaves, respectively, *P* = 0.1636).

### Root diameter

**Figure 1:**
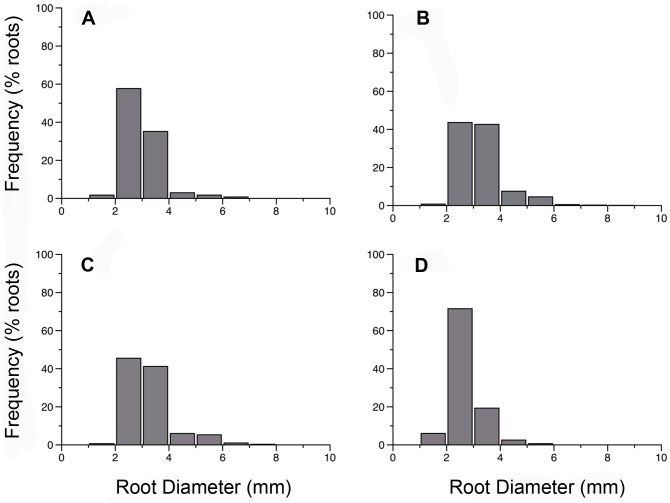
Frequency distribution of root diameters (mm) for first and second order roots of (A) *L. japonica*, (B) *L. sempervirens*, (C) *F. alnus* and (D) *R. alnifolia*.

For all four species, observed first and second order root diameters were 1.01 mm to 8.4 mm, with most root diameters falling between 1–3 mm (Fig. 1, Fig. S2). Average diameter of first and second order roots of *L. japonica* was smaller in comparison to *L. sempervirens* (2.84 mm, n = 404, 3.18 mm, n = 784, respectively, *P*<0.01). *F. alnus* had larger average root diameters for first and second order roots compared with *R. alnifolia* (3.10 mm, n = 301, 2.60 mm, n = 316 respectively, *P*<0.0001).

### Total and seasonal root production

Among all four species, native species had a higher total root production of first and second order fine roots compared to non-native congeners between July 2010 and February 2012.

*L. sempervirens* had the highest total production over the 704 days (794 roots, 43.3%, Table 1), although differences between *Franugla* and *Rhamnus* species were not as significant. The overall highest seasonal root production across all four species occurred during summer (June to August), an average of 53.3% (Table 1) of the total roots produced and fall (September to November) with an average of 26% (Table 1). The fewest roots overall grew during winter (December to February) with an average of 4.6% of the total roots produced.

**Table 1:** Resutls of Chi-square test for differences in total first and second order fine root production between congeneric pairs of *Lonicera* and Rhamnaceae.

Species	No. roots/season (Sp, Sm, Fa, Wn*)	Proportion of total (%)	Total no. roots	χ^2^	*P*
*L. japonica*	109	26.6	410		
	134	32.7			
	142	34.6			
	25	6.1			
*L. sempervirens*	132	16.6	794	48.3	<0.0001*
	425	53.5			
	199	25.1			
	38	4.8			
*F. alnus*	22	7.2	307		
	207	67.4			
	70	22.8			
	8	2.6			
*R. alnifolia*	30	9.3	321	2.71	0.43
	211	65.7			
	66	20.6			
	14	4.4			
**Total**	293	16	1832		
	977	53.3			
	477	26			
	85	4.6			

* (from top to bottom) Sp = spring (March-May), Sm = summer (June-August), Fa = Fall (September-November, Wn = Winter (December-February).

When comparing the non-native versus the native species of the two genera, fine root production was significantly different in total and seasonal first and second order fine root production (Table 1). *L. sempervirens* produced in total more fine roots than *L. japonica* (*P<0.0001*), while the latter produced a higher proportion of fine roots relative to *L. sempervirens* in spring (26.6% versus 34.6%) and fall (16.6% versus 25.1%) respectively, In Rhamnaceae, seasonal fine root production differed less (*P* = 0.438), although *R. alnifolia* produced more roots in spring (67.4% versus 22.8%) and winter (65.8% versus 20.6%) and *F. alnus* produced more roots during summer and fall, although these differences were not significant (*P*>0.05).

### Root standing crop

**Figure 2:**
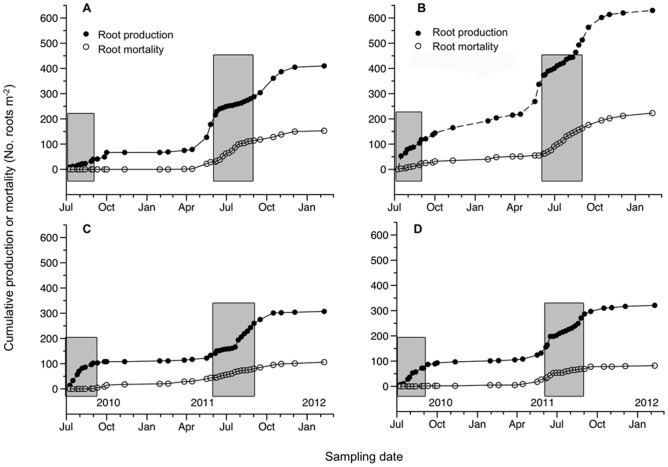
Cumulative root production and mortality for (A) *L. japonica*, (B) *L. sempervirens*, (C) *F. alnus*, (D) *R. alinfolia*. Closed circles (•) represent cumulative root production as the number of roots per m^2^, and open circles (○) represent cumulative root mortality as the number of roots per mm^2^. The number of roots produced for *L. sempervirens* were adjusted to zero. Gray bars highlight the summer season (June-August).

*L. sempervirens* had a higher root standing crop (root biomass) compared to *L. japonica* (Fig. 2A & B, Fig. S3), due to the high number of roots viewed on the first day of observation. *L. sempervirens* showed higher root mortality during fall and winter, whereas *L. japonica* had higher root mortality during late spring and summer.

Root standing crop did not differ between *Rhamnus* species (Fig. 2C & D, Fig. S3). However, *R. alnifolia* had higher fine root mortality over the summer months, while the *F. alnus* had consistent mortality across summer, fall, and winter, and low mortality during spring.

### Root lifespan

The following covariates had a significant influence on root lifespan in *Lonicera* species: species, root order, and the number of neighboring roots at the time of root death (Table 2). The native species *L. sempervirens* had a 180.8% ([2.808–1]×100%) longer fine root lifespan in comparison to non-native *L. japonica* (*P*<0.0001), although both species had more than 50% fine root survivorship at the end of the study period. Lifespan of first order roots decreased by 215% compared with second order roots (*P*<0.0001), and lifespan of roots decreased by 171.8% ([e^0.2496^–1]×100%) with an increase in each number of neighboring roots at the time of root death. The same covariates, which influenced fine root lifespan of *Lonicera*, significantly influenced root lifespan of the *Frangula* and *Rhamnus* species (Table 2). Fine root life span of the non-native *F. alnus* was 35.3% shorter compared with native *R. alnifolia* (*P* = 0.0036). Root lifespan increased by 74% from first to second order roots (*P* = 0.011), and decreased by 172% for each additional neighboring root present at the time of root death (*P*<0.0001). Root diameter did not have a significant influence on root life span for either Rhamnaceae or *Lonicera* species (*P* = 0.839, *P* = 0.059, respectively).

**Table 2:** Proportional hazards regression analysis results for root life span of congeneric natives (*L. sempervirens* and *R. alnifolia*) and non-natives (*L. japonica* and *F. alnus*).

Variable	df	Parameter estimate	Std. error	χ^2^ value	P-value	Hazard ratio
***Lonicera***						
Neighbor	1	0.250	0.012	408.89	**<0.0001**	1.283
Species						
*L. japonica*	1	1.033	0.112	84.87	**<0.0001**	2.808
Root order		-	-	26.16	**<0.0001**	-
First order	1	1.147	0.224	26.16	**<0.0001**	3.15
Diameter	1	-	-	3.57	0.059	-
**Rhamnaceae**						
Neighbor	1	0.289	0.020	217.13	**<0.0001**	1.335
Species		-	-	8.49	**0.004**	-
*R. alnifolia*	1	-0.436	0.150	8.49	**0.004**	0.647
Root order		-	-	6.55	**0.011**	-
First order	1	0.554	0.216	6.55	**0.011**	1.74
Diameter	1	-	-	0.041	0.839	-

*Significant values are denoted in bold.

*Non-significant parameters were removed from the regressions model.

**Figure 3:**
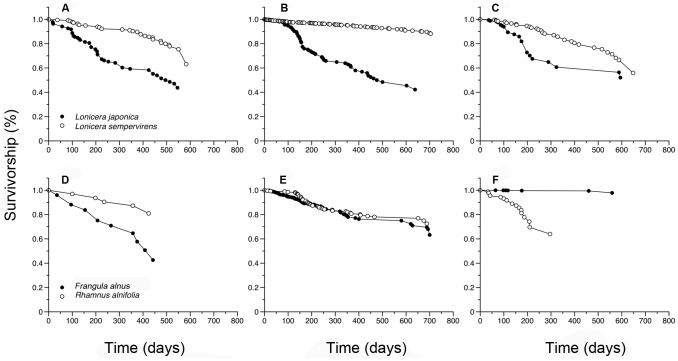
Fine root survivorship of native and non-native congeners *L. japonica*, *L. sempervirens* (A–C) and *F. alnus*, *R. alnifolia* (D–F) over roots born during spring (A, D), summer (B, E) and fall (C, F) seasons. Data are for all roots observed from July 2010 through October 2011. Open circles (○) indicate non-native congeners and black circles (•) represent natives.

Fine roots of *L. sempervirens* born in spring, summer, and fall were significantly longer-lived compared to fine roots of *L. japonica* (*P*<0.0001, Fig. 3A–C). Fine roots of the non-native *L. japonica* born in spring and summer reached a median survivorship of 495 days and 476 days, respectively, while fine roots born during fall only reached a survival at the 75^th^ percentile of 213 days. No significant difference in median lifespan of fine roots of the non-native *L. japonica* was found for roots born during spring and summer (*P* = 0.468). Fine roots of *L. sempervirens* only reached the first quartile of survivorship at 551 days for roots born in spring and 516 days for roots born in fall. Estimations for fine roots born during the summer show that 88% remained visible for more than 551 days.

Differences in fine root lifespan between *Frangula* and *Rhamnus* species were detected in spring and summer (Fig. 3D–F). Fine roots of native *R. alnifolia* were significantly longer-lived when born in the spring (*P* = 0.007) and summer season (*P* = 0.002) compared to non-native *F. alnus*. While fine roots of *R. alnifolia* were shorter-lived compared to *F. alnus* during the fall season, the difference in lifespan was not significant (*P* = 0.299). Fine roots of non-native *F. alnus* reached median survival time of 408 days for roots born during spring, the 75^th^ percentile at 580 days for roots born during the summer, and reached 97% survivorship at 559 days for roots born during the fall, indicating an increase in lifespan across seasons from spring to fall. Fine roots of native *R. alnifolia* reached a final survivorship of 80% at 675 days for roots born during spring, 75^th^ percentile survival at 675 days for roots born during the summer, and the 75^th^ percentile survival at 208 days for roots born during the fall. Roots of *R. alnifolia* were longest-lived during the spring season, and shortest-lived during the fall.

### Relationship between leaf and root production and lifespan

In general, both non-native species exhibited higher aboveground leaf production and lower root production within a single growing season. Average leaf lifespan lasted between 38 days to 120 days. Root and leaf lifespan had the same pattern of shorter-lived tissue in non-native *L. japonica* and longer-lived tissue in native *L. sempervirens*. In Rhamnaceae species, non-native *F. alnus* had shorter-lived roots, although average leaf lifespan was similar between the two species.

### Seasonal N uptake

Significant differences were observed with time of uptake, season and the interaction between time and season (*P*<0.0001, Table 3). The parameters that contributed to significant increases in ^15^N content were during the six-hour time period (*P*<0.0001) and the interaction of season and time at six hours (*P*<0.0001). Uptake was significantly higher during spring than the fall season (*P*<0.0001, Table 3). For spring and fall, there were no differences in ^15^N uptake between native and non-native species within either Rhamnaceae and *Lonicera* (*P* = 0.233).

**Table 3:** Results from a mixed-effects model testing native status, season, time and the interaction of season and time across 15N concentrations in roots of *L. japonica*, *L. sempervirens*, *F. alnus*, and *R. alnifolia*.

Factor	DF	Parameter Est	St. Err.	t	*P>*|*t*|
Native status	1				
Non-native		0.0009	0.0005	1.7	0.233
Time	3				
T(0)		−0.001	0.0005	−1.85	0.066
T(3)		−0.0003	0.0005	−0.57	0.572
T(6)		0.003	0.0005	5.28	**<0.0001**
Season	1				
Fall		−0.002	0.0003	−6.91	**<0.0001**
Season*Time	3				
Fall*T(0)		0.0008	0.0005	1.56	0.119
Fall*T(3)		0.0004	0.0005	0.85	0.395
Fall*T(6)		−0.002	0.0005	−4.66	**<0.0001**

Significant differences are in bold. T(x)  =  time (hours).

15 N concentrations are measured in [µg 15N µg root tissue^−1^].

**Figure 4:**
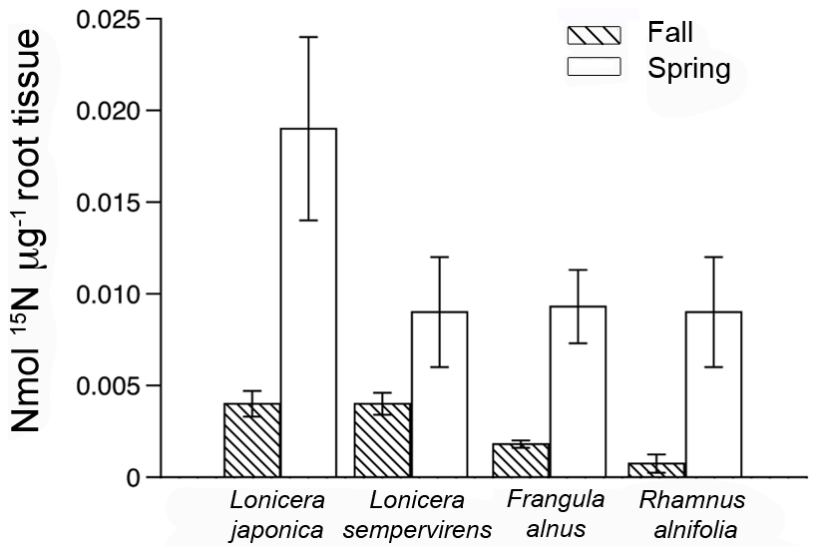
Mean ± SE of ^15^N (nmol ug^−1^ root tissue) accumulated in new first and second order fine roots during fall 2011 and spring 2012 at 6 hours of exposure to 1 mmol ^15^N exposure. Species are ordered from left to right: *L. japonica*, *L. sempervirens*, *F. alnus*, *R. alnifolia*.

When the analysis was separated by individual season, roots born in the spring had approximately 100 times higher nitrate concentrations after ^15^N exposure than roots from the fall (Fig. 4, Table 3). During spring uptake, less than 0.01 mmol KNO_3_^-^ was accumulated in the tissue until hour six, where a significant increase in uptake occurred (*P*<0.0001). Root initial contact time with nitrate was significant with a comparatively lower level of uptake (*P* = 0.042), so its effect on total uptake was minimal overall. Roots of non-native species had a mean nitrate content of 0.008±0.0007 mmol ^15^N µg^-1^ root tissue, which was significantly higher than native congeners (*P* = 0.009). No significant effect of time or native status was found during fall uptake (*P*>0.05), but there was a lower uptake trend in native plants (Fig. 4).

## Discussion

Using two congeneric pairs of native and non-native understory plants with vastly contrasting leaf phenology, we investigated their hidden dynamics of fine root growth in comparison to leaf dynamics, carbon acquisition and distribution. Our results indicate links between belowground and aboveground resource-related traits that, together, elucidate growth strategies of non-native species, underlying their invasive advantages.

For *Lonicera* species, non-native *L. japonica* had flushes of root production earlier in the spring and later in the fall, conferring a potential advantage for extended periods of nutrient acquisition. We expected the timing of root flushes would follow the onset of spring leaf production, for measurements within the same year's root growth, regardless of root and leaf phenology measurements taken across separate years [45], [46]. Potential reasons for root production to follow leaf production could be related to the earlier warming of the atmosphere relative to soil temperatures at the start of the growing season associated with leaf production [46] or root growth dependency on aboveground carbon fixation [45], [54]. Additionally, previous findings showed that stored photosynthate is used to initiate fine root growth in higher plants before leaf development in woody trees [46]. Still, understory plants are usually first to develop and last to lose vegetation seasonally compared to over-story trees in temperate regions, providing carbon stores needed for extended root production [8], [9].

Temporal asynchrony between fine root production and leaf production was seen between both congeneric pairs of species. *Lonicera* vines had the highest periods of fine root production during the spring and fall, while the highest production of leaves occurred during the summer. In Rhamnaceae shrubs, fine root production was highest during the summer, while leaf production was greatest during the spring. This pattern fit the hypothesis proposed by Pregitzer *et al*. [55] that species partition the growth of above and belowground tissues because foliage and fine roots may be competitive sinks for C. The differences in timing between fine root and leaf production in *Lonicera* and Rhamnaceae is most likely due to growth form. Since vines, unlike shrubs, do not require the same amount of mechanical support for vertical growth, they can initially allocate more to root production rather than increasing stem thickness after initial leaf production, as well as allocate carbon towards summer leaf production and stem length for water transport [56], [57].

Non-native *L. japonica* had lower overall fine root biomass compared with native *L. sempervirens* (Fig. S3). However, biomass alone in *Lonicera* vines may not be a suitable indicator for the advantage of non-natives over native species. Others have found that native *L. sempervirens* had higher aboveground biomass accumulation than *L. japonica* in the absence of herbivores, suggesting that annual carbon gain, as an independent factor, does not confer an advantage for this species [58]. In the presence of herbivores, *L. japonica* showed a compensatory response of increased aboveground growth [58]. If similar to other non-native species that exhibit a compensatory response to aboveground herbivory, defoliation events may decrease root and total biomass, yet increase N uptake from roots and N remobilization from leaves to compensate for increased N demand, leading to short-term growth advantages [59].

In addition to differences in fine root production, we also found a strong difference in fine root lifespan between *Lonicera* species (*P*<0.0001). Unlike Withington *et al*. [47] who found no correlation between aboveground and belowground traits among 11 temperate tree species, we found a corresponding pattern in leaf and fine root life span, with non-native *L. japonica* exhibiting significantly shorter fine root life span and leaf lifespan compared with native *L. sempervirens*. Seasonally, fine roots exhibited increasing lifespan from spring to fall in *L. japonica* and life span was variable in *L. sempervirens*, though lowest in the spring. This finding from our study was consistent with other studies of root dynamics in temperate forests, where fine root production is highest and lifespan is shortest during warmer seasons. In this case increased soil nutrient availability from higher rates of soil microbial decomposition, high photosynthetic and respiration rates can decrease tissue construction costs and increase fine root turnover, lowering fine root lifespan [37], [52], [60]. However, our results did not corroborate our hypothesis of correspondingly higher root tissue production and turnover throughout the fall as non-natives continued extended leaf production. A shorter root life span across seasons in non-native *L. japonica* suggests higher turnover with important implications of more effective resource capture, with the age of roots having a central role in the time period roots are physically active [53], [61]. The capacity for seasonal fine root N acquisition in individual fine roots of *L. japonica* was demonstrated through higher ^15^N uptake (Fig. 4), though fall uptake did not suggest extended seasonal N uptake to support extended leaf phenology.

In contrast to *Lonicera* species, we found no differences between *Frangula* and *Rhamnus* species in total and seasonal root production or root mortality to support our hypothesis of a link between extended leaf phenology and fine root growth, despite strong contrasts in aboveground phenology and known differences in growth rate (Fig. S1) [62]. We did find, however, that native *R. alnifolia* differed from non-native *F. alnus* in the timing of beginning seasonal root growth and earlier leaf drop. For understory species, early leaf production before canopy closure followed by allocation of photosynthates belowground may be critical to periods of low light during canopy closure and dormant winter months [63]. Additionally, in accordance with our hypothesis, native *R. alnifolia* exhibited significantly longer root survivorship than non-native *F. alnus* (Table 2), similar to the longer root survivorship of native *L. sempervirens*. This fact also supports the association between fine root tissue longevity, tissue N concentration, growth rate, and invasive potential [26]. [62], [64]–[66]. Although no distinct pattern in seasonal root life span was found between species, the small number of uncensored roots during spring and fall seasons may have been a contributing factor to the results, warranting a longer period of observation. While not significant, there was a trend in greater spring N uptake in fine roots of *F. alnus* compared with *R. alnifolia*, suggesting a higher capacity for ephemeral resource capture (Fig. 4). Shorter root lifespan may be potentially linked with higher uptake of N in spring.

We analyzed multiple covariates contributing to the risk of root mortality because of the long fine root life span observed. A consistent risk of root mortality was the covariate root order across all four species, where first order roots were shorter lived than higher second order roots (Table 2). This is in line with similar results showing that root branching order had the strongest effect on root life span [35]. However, the lack of influence of root diameter on root mortality from both pairs of congeneric species was contrary to the conclusions from Wells and Eissenstat [52] and McCormack *et al*. [38] on the strength of root diameter as a predictor of lifespan. The strength of prediction in longevity between root orders may be a result of differences in fine root anatomy of the categorical orders within the root hierarchical structures, while arbitrary classification based on diameter may include multiple orders of roots [32], [67], [68]. Likewise, due to the branching structure of roots, if higher order roots die then more distal, lower order roots must also die, making order a more precise way to categorize fine roots. This is especially the case when considering that root order persistence observed by the minirhizotron technique includes also the time to decompose fine root structures when the opposite trend was revealed that dead fine roots of lower order possibly persisting longer than higher order fine roots [30], [69].

We did not measure mycorrhizal colonization, which has been reported as a factor increasing root life span [36]. Mycorrhizal colonization of the lowest order roots consisting of primary development may be associated with increased protection against pathogens and increased nutrient acquisition [70]. Even so, mycorrhizal colonization can decrease the content of phenolic defense compounds in fine roots [71]. While species of both Rhamnaceae and *Lonicera* genera associate with arbuscular mycorrhizal (AM) fungi, the benefits of mycorrhizal colonization in these species have not been studied, though AMF have been found to foster successful invasions by other non-native plants in riparian habitats [72].

We also demonstrated that non-native species differed in growth strategies, with non-native *L. japonica* showing higher biomass allocation to aboveground leaf production while the native *L. sempervirens* demonstrated the opposite, a higher belowground fine root production, along with differential timing of tissue production between *Frangula* and *Rhamnus* species. These differences may be due in part to resource use strategies employed by non-native species, which typically exhibit disproportionate bias towards aboveground growth under high light conditions [73]. Higher biomass allocation to aboveground leaf production in non-natives may stem from the need to capture light resources in the understory before canopy closure. Non-native invasive congeners also showed capacity for high root turnover, associated with increased N uptake, paralleling the capacity for fast growing leaf foliage to capture higher amounts of C [53], [65]. Future experiments addressing same-year resource partitioning to above- and belowground tissues may provide further insight into whole-plant strategies for successful resource capture strategies.

## Supporting Information

Figure S1 **Leaf demography of A)** ***L. japonica*** **, B)** ***L. sempervirens*** **, C)** ***R. frangula*** **, and D)** ***F. alnus*** **growing in a common garden over the 2008 growing season.**
(TIF)

Figure S2 **Average root diameter (mm) of first and second order roots of** ***L. japonica*** **,** ***L. sempervirens*** **,** ***F. alnus*** **and** ***R. alnifolia*** **.**
(TIF)

Figure S3 **Cumulative root standing crop of congeneric** ***L. japonica*** **,** ***L. sempervirens*** **,** ***F. alnus*** **, and** ***R. alnifolia*** **.**
(TIF)
