# Supplementary figures and images for: Links between Belowground and Aboveground Resource-Related Traits Reveal Species Growth Strategies that Promote Invasive Advantages
Source: PLoS One. 2014 Aug 8;9(8):e104189. doi: 10.1371/journal.pone.0104189 (PMC4126695; doi:10.1371/journal.pone.0104189)

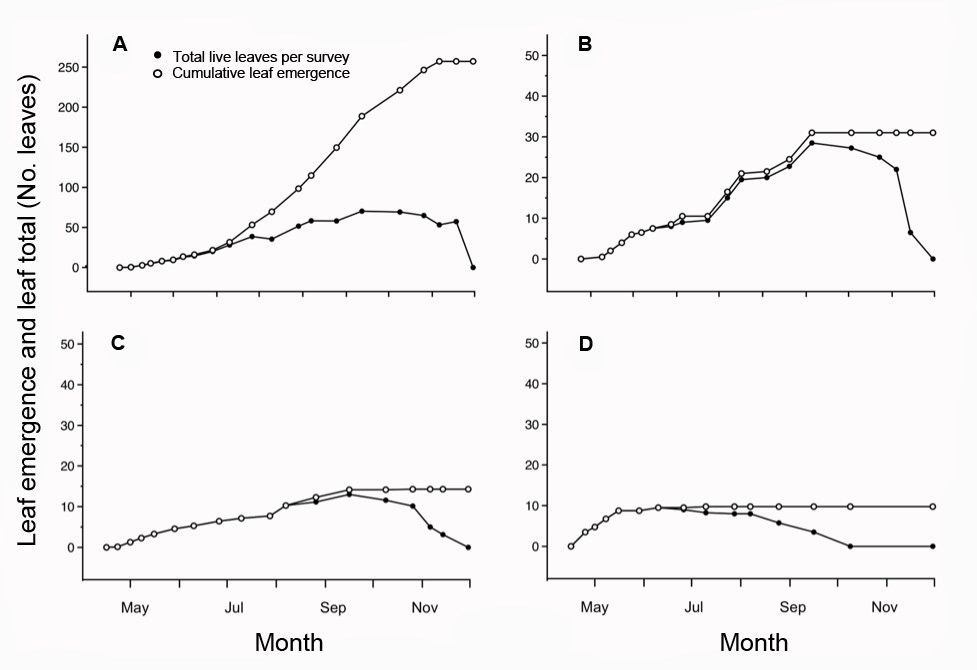

Supplement: Figure S1 — Leaf demography of A) L. japonica , B) L. sempervirens , C) R. frangula , and D) F. alnus growing in a common garden over the 2008 growing season. (TIF) [file pone.0104189.s001.tif]

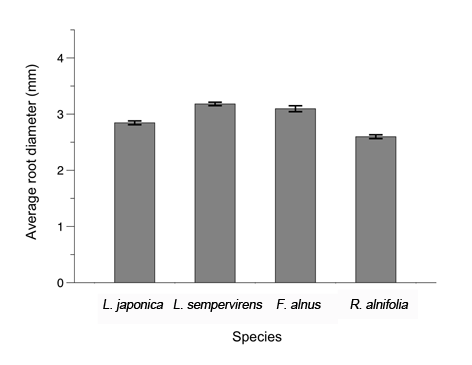

Supplement: Figure S2 — Average root diameter (mm) of first and second order roots of L. japonica , L. sempervirens , F. alnus and R. alnifolia . (TIF) [file pone.0104189.s002.tif]

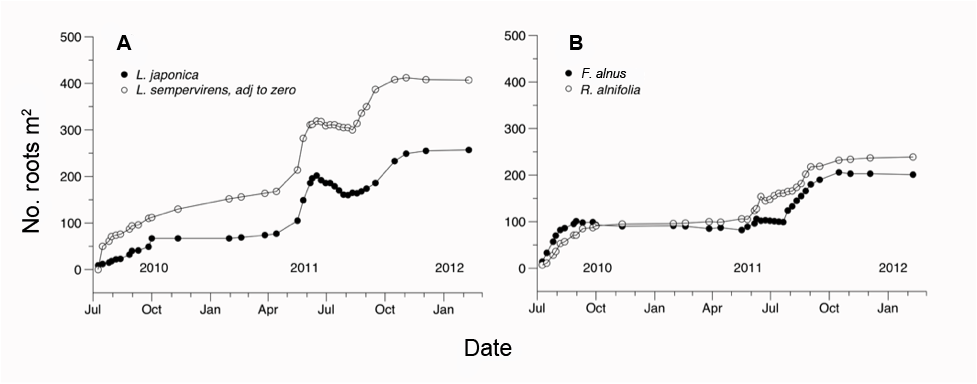

Supplement: Figure S3 — Cumulative root standing crop of congeneric L. japonica , L. sempervirens , F. alnus , and R. alnifolia . (TIF) [file pone.0104189.s003.tif]
